# Pain perception following computer-controlled versus conventional dental anesthesia: randomized controlled trial

**DOI:** 10.1186/s12903-022-02454-1

**Published:** 2022-09-22

**Authors:** Sameh Attia, Thomas Austermann, Andreas May, Mohamed Mekhemar, Jonas Conrad, Michael Knitschke, Sebastian Böttger, Hans-Peter Howaldt, Abanoub Riad

**Affiliations:** 1 Department of Oral and Maxillofacial Surgery, Justus-Liebig-University, Klinikstrasse 33, 35392 Giessen, Germany; 2 Clinic for Conservative Dentistry and Periodontology, School of Dental Medicine, Kiel University, Arnold-Heller-Str. 3, Haus B, 24105 Kiel, Germany; 3 Department of Public Health, Faculty of Medicine, Masaryk University, Kamenice 5, 625 00 Brno, Czech Republic; 4 Czech EBHC: JBI Centre of Excellence, Institute of Biostatistics and Analyses, Faculty of Medicine, Czech National Centre for Evidence-Based Healthcare and Knowledge Translation (Cochrane Czech Republic, Masaryk University GRADE Centre), Masaryk University, Kamenice 5, 625 00 Brno, Czech Republic

**Keywords:** Computed-controlled local anesthesia, Dental anesthesia, Dental education, Local anesthesia, Nerve block, Pain perception, RCT, Split-mouth

## Abstract

**Background:**

The administration of local anesthesia (LA) in dental practice requires an injection which is the leading cause of patients’ fear and anxiety. Computer-controlled local anesthetic injector, designed to reduce the pain of performing local anesthesia by controlling the speed of injection. This single-blind randomised control trial aimed to compare the pain perception after computer-controlled local anesthesia (CCLA) and conventional LA.

**Methods:**

Dental students were both test and operator group versus an experienced dentist as additional operator of the LA. Data were collected regarding gender, age, medical condition, smoking habits. Additionally, operator feedback about the handling, pain at insertion and during infiltration, excitement (Dental Anxiety Scale), and complications were assessed.

**Results:**

Out of the 60 included participants, the majority were females (*n* = 41; 68.3%), medically healthy (*n* = 54; 90%), and did not receive medications (*n* = 54; 90%). While the participating students administered 62 (51.7%) injections, the experienced dentist administered 58 (48.3%) injections. The difference in pain perception on puncture between CCLA and conventional injections was not statistically significant (*Sig*. = 0.285); however, pain perception during injection was significantly different (*Sig*. = 0.029) between CCLA (1.65 ± 1.93) and conventional injections (2.49 ± 2.31).

**Conclusion:**

The professional experience influenced the pain perception while applying the LA. CCLA did not reduce pain on puncture significantly; however, pain perception during the injection was significantly reduced in the case of using CCLA devices compared to the conventional syringe.

## Background

Since the first described use of local anesthesia (LA) in 1884, the goal of pain-free or pain-reduced treatment in dentistry has been achievable [1]. Several treatment procedures in clinical dentistry and oral and maxillofacial surgery are associated with pain, for which the adjuvant of LA is essential in terms of pain reduction [2]. In the USA, about 300 million cartridges of LA have been applied annually [3, 4]; in Germany, an estimated 70 million carpels are used annually [5], and in Australia, this figure is about 11 million [6]. Dentists often give more than 1,500 cartridges of LA every year to their patients [7]. This magnitude demonstrates LA relevance in the fields of dentistry and oral and maxillofacial surgery [8].

The administration of LA in dental practice requires an injection which is the leading cause of patients’ fear and anxiety [9]. The failure to provide adequate pain management can be a significant source of worry for dental practitioners [10]. Continuous developments at the pharmacological level, e.g., active substances, and the technical level, e.g., the application form, should enable optimisation of the sufficient LA use [4, 11]. The associated adverse effects such as injection pain, the latency of effects onset after injection, risks associated with accidental intravascular injection require further research for improvement [11]. Therefore, dental students -as future dentists- need to be well-trained to administer efficient and safe dental LA. Additionally, dental anesthesia educational courses should include all modern local anesthesia equipment in order to provide up-to-date dental education and improve the dental students’ LA training skills [12, 13]. This includes also the pre-anesthesia approach by using topical anesthetic on the needle insertion site, which has been proven to reduce the needle insertion pain in inferior alveolar nerve block [14].

One aspect of this advancement is the launch of a computer-controlled local anesthetic injector Calaject^®^, (Rønvig Dental MFG, Daugaard, Denmark), designed to reduce the pain of performing local anesthesia [15]. The principle of this device is based on the fact that the less pressure and flow of a local anesthetic injection, the less painful the procedure will be [16]. Each device has an installed pressure sensor as well as a three-button display that allows you to choose the most appropriate program in terms of different speeds and pressure. According to the anesthesia technique, the manufacture recommends program I for intraligamentary and palatinally injections, program II for infiltration, and III for alveolar nerve block techniques. Conventional carpules and needles can be used in a pen-shaped part connecting to the main unit. The anesthetic administration can be achieved using a foot control pedal adapted to the main unit. The speed of injection is related to acoustic signals [17].

Evaluating the effectiveness of the computer-controlled local anesthesia (CCLA) compared to the conventional methods in a clinical trial setting is essential and required in the literature [18, 19]. Dental students as the test and operator group and an experienced dentist as an additional operator can give a better understanding of whether the dentist’s experience plays a role in terms of pain perception and demographic and medical risk factors [20]. To the best of the authors’ knowledge, this is the first trial that used this setting with two operator’s “students” and “dentist”. As we apply the CCLA and conventual LA in the dental education (student-to-student) and correlate the result with applying the two methods by an experienced dentist. Additionally, we consider two types of pain in this study: the first at the puncture site and the second during the application.

Pain perception may vary between male and female [21, 22], smoker and non-smoker [23, 24] ,and healthy and diseased patients [25]. Several studies reported a correlation between dental anxiety and pain perception in different dental treatments [26–28]. An evaluation of pain perception by applying LA in both CCLA and conventional form would help develop a gender/ patients-specific LA administration technique.

## Methods

### Aim

This study aimed to compare the pain perception using conventional dental anesthesia to the computer-controlled device Calaject^®^ as a part of the dental local anesthesia curricula. The focus was on the comparison of the parameters by doing the two types of LA by dental students and experienced dentist to find out whether the dentist experience play any role on the pain level.

### Trial design

**Fig. 1:**
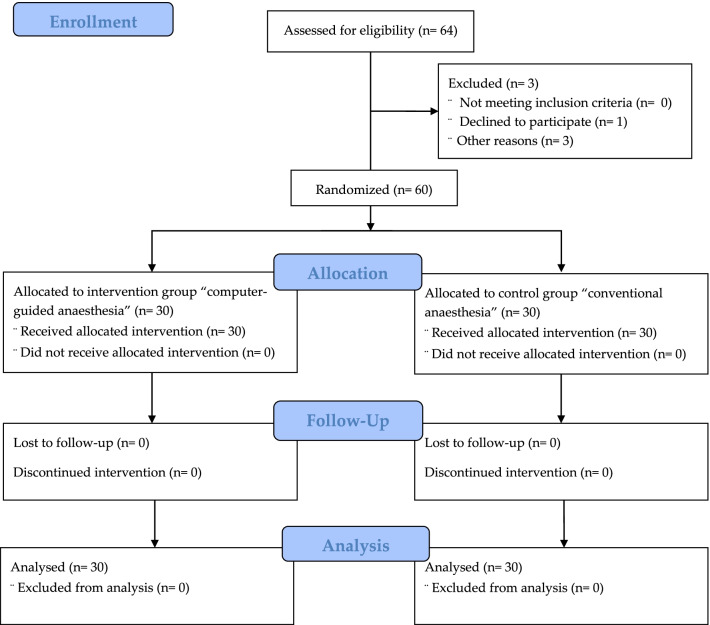
CONSORT 2010 Flow Diagram

This study was designed as a single-blind randomised controlled trial (RCT) that is reported in compliance with the Consolidated Standards of Reporting Trials (CONSORT) statement of 2010 [29]. The CONSORT 2010 flowchart in Fig. 1 highlights the workflow of the study, which started with assessing the eligibility of 64 subjects, four of whom were either excluded or withdrawn before the randomisation phase. The interventions were equally and randomly distributed between the two study groups: experimental group (computer-controlled anesthetic injection) and control group (conventional anesthetic injection). No intervention event was lost during the following nor excluded during the outcome analysis.

### Participants

The results of this study were generated as part of the dental training during the anesthesia technique course at Justus Liebig University, Giessen, Germany. Two methods were available to apply the local anesthetic (0.5 ml, Ultracain D without vasoconstrictor, Sanofi-Aventis, Frankfurt, Germany): (1) conventional injection using Uniject syringe, (2) computer-controlled injection using Calaject system^®^ [30]. Identical needle diameters (Painless Steel^®^, Disposable injection cannulas, 7G − 0.4 × 23 mm, Transcodent^®^, Sulzer Management Ltd, Winterthur, Switzerland) were used for both systems. The participants were dental students who were motivated to participate in this study as a part of their LA course. Regarding the operator there were two groups; (a) the “students” group in which students gave each other the LA and (b) the “dentist” group in which an experienced dentist was the operator and the dental students were the study subjects.

The students were instructed to inject the LA in the conventional art slowly as the doctrine defined the injection time as one ampule per minute. The dentist applies the conventional LA in the same speed to have better comparability.

The “student-administered” group included dental students in their 3rd year of training, who administered conduction anesthesia in the lower right inferior alveolar nerve and infiltration anesthesia in the region of the first right premolar to each other. The “dentist-administered” group “dentist” included infiltration anesthesia in a “split-mouth” model of the same subject in region first premolar left and right by means of both types of injections by an experienced oral surgeon. As it was not a part of the training course, we decided to make an infiltration in a split-mouth design for better comparability between the computer-controlled and conventional LA. All infiltration LA was applied in the buccal side of the first premolar in the non-keratinized mucosa (Table 1).

**Table 1:** Characteristics of the study groups according to the operator

Study groups	Student-administered	Dentist-administered
Operator	Students (n = 31)	Dentist (n = 1)
Subjects	Students (n = 31)	Students (n = 29)
Injection Techniques	Supraperiosteal Infiltration (first right premolar) (n = 31) Inferior Alveolar Nerve Block (right side) (n = 31)	Supraperiosteal Infiltration (first right and left premolar) (n = 58) Inferior Alveolar Nerve Block (n = 0)
Injection Methods	Computer-controlled (n = 31):16 IAN-B and 15 Infiltration Conventional (n = 31):15 IAN-B and 16 Infiltration	Computer-controlled (n = 29) Conventional (n = 29)
Randomization	The injection methods were simply randomized between the two injection techniques (even: computer-controlled /odd: conventional)	The injection methods were simply randomized between the right and left injection sites (even: computer-controlled /odd: conventional)
Blinding	The students were blinded to the injection methods	The students were blinded to the injection methods
Split-mouth	Yes	No

### Sample size

**Fig. 2:**
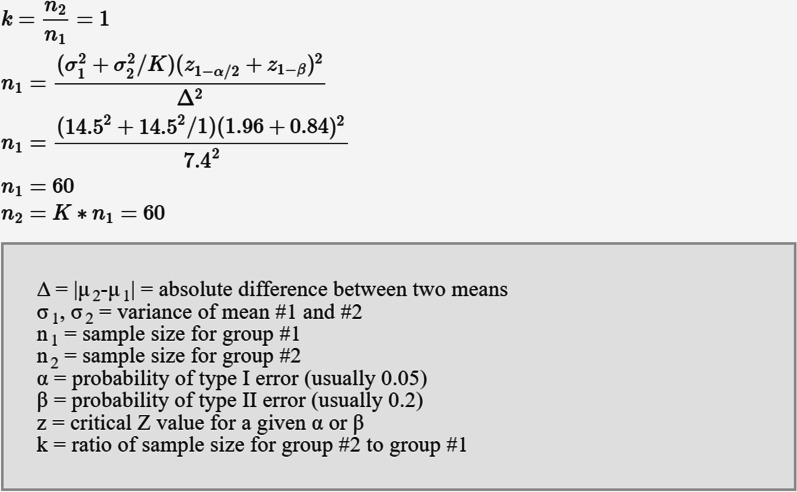
Sample Size Calculation, ClinCalc (ClinCalc LLC., Chicago, IL, USA)

The sample size required for this study was calculated using ClinCalc (ClinCalc LLC., Chicago, IL, USA) based on the outcomes of previous literature [31–33]. The enrollment ratio was set to be 1:1, the alpha error (α) was ≤ 5%, and the test power (1-β) was 80%. Therefore, the pragmatic sample size was 120 interventions, including 60 experimental (computer-controlled) and 60 control (conventional) (Fig. 2).

### Study parameters

Data were collected regarding gender, age, medical condition, smoking habits. Additionally, operator feedback about the handling (conventional versus computer-assisted injection), pain at insertion and during infiltration (category: “none”, “mild”, “moderate”, “severe”, and numerical/scaled), excitement (Dental Anxiety Scale), and complications (yes vs. no) were assessed. The assessment of pain in both puncture site and during the injection was documented immediately after the injection was performed to obtain an accurate record. The students were given five minutes as washout time.

A visual analog scale (VAS) was used to assess the pain severity as the validity of this method for pain measurement was previously proved [34]. The dental Anxiety Scale (DAS) [35] was also applied for all study subjects to exclude any excessive pain sensation due to dental anxiety.

### Randomization and blinding

Unilateral blinding was performed beforehand for the purpose of application type and location. As the computerised injection system emits a signal tone, this signal tone was during the application of the conventional local anesthesia. During the procedure, the subjects were deprived of their sense of sight by means of a blindfold. The selection of the application sequence used (computerised vs. conventional) was randomised to avoid an expectation from the study subject to the type of LA used.

### Ethical considerations and registration

This study was carried out in accordance with the Declaration of Helsinki for research involving human subjects [36]. The study protocol was thoroughly reviewed and approved by the Ethics Committee of Justus Liebig University of Giessen Ref. 77/19 on 07 May 2019 and retrospective registered in the ClinicalTrials.gov clinical database (Identifier: NCT05192902) on 14.01.2022. Written informed consent was obtained from all the study participants before their participation, and the participants were able to withdraw from the study at any stage without justification. Participation was voluntary, and no disadvantages resulted from non-participation, and no financial incentives or inducements were offered for participation.

### Statistical analysis

The Statistical Package for the Social Sciences (SPSS) version 27.0 (SPSS Inc., Chicago, IL, USA, 2020) was used to perform all statistical tests [37]. Descriptive statistics were executed to summarise the characteristics of the participants (gender, medical anamnesis, and smoking), the interventions (operator, injection technique, and method), and the outcomes (feedback, pain perception, and DAS) using frequency (*n*) and percentage (%) for categorical variables and mean (*µ*) and standard deviation (*SD*) for numerical variables. Shapiro-Wilk test was used to check VAS and DAS score for normal distribution (Sig. ≤ 0.05).

Consequently, inferential statistics were executed to test the association between the participants’ characteristics and their feedback, pain perception, and DAS score. Bivariate correlation was performed to evaluate the correlation between DAS and VAS scores, and the correlation coefficients were interpreted according to Chan’s methodology; as 0 = no correlation, 0.1–0.2 = poor correlation, 0.3–0.5 = fair correlation, 0.6–0.7 = moderate correlation, 0.8–0.9 = strong correlation, and 1 = perfect correlation [38]. All inferential tests were performed assuming a confidence level (*CI*) of 95% and a significance level (*Sig.*) of ≤ 0.05.

## Results

### Sample characteristics

Out of the 60 included participants, the majority were females (*n* = 41; 68.3%), medically-free (*n* = 54; 90%), and do not receive medications (*n* = 54; 90%). Smoking was uncommon among the participants (*n* = 4; 6.7%), with three students consuming 1–5 cigarettes per day and one student consuming 6–15 cigarettes per day (Table 2).

**Table 2:** Demographic, medical and behavioral characteristics of the participants (*n* = 60)

Variable	Outcome	Frequency (*n*)	Percentage (%)
Gender	Female	41	68.3
	Male	19	31.7
Chronic Illnesses	Yes	6	10
	No	54	90
Medical Treatments	Yes	6	10
	No	54	90
Smoking	Yes	4	6.7
	No	56	93.3
Smoking Frequency	None	56	93.3
	1–5 cigarettes/day	3	5
	6–15 cigarettes/day	1	1.7
	> 15 cigarettes/day	0	0

### Intervention characteristics

While the student participants administered 62 (51.7%) injections, the experienced dentist administered 58 (48.3%) injections. Most injections were administered as Supraperiosteal infiltration (*n* = 89; 74.2%) and the rest consisted of inferior alveolar nerve blocks (*n* = 31; 25.8%). The administered injections were equally distributed over the study arms, as 60 (50%) were computer-controlled and 60 (50%) were conventional (Table 3)

**Table 3:** Characteristics of the anesthetic injections received and administered by the participants (*n* = 120)

Variable	Outcome	Frequency (*n*)	Percentage (%)
Operator	Student	62	51.7
	Dentist	58	48.3
Injection technique	Supraperiosteal Infiltration	89	74.2
	Inferior Alveolar Nerve Block	31	25.8
Injection method	Computer-controlled	60	50
	Conventional	60	50

### Operator’s feedback

On asking the operator students about their feedback with administering the anesthetic injection, 26 (41.9%) preferred the computer-controlled method, 28 (45.2%) preferred the conventional method, and 8 (12.9%) rated both equally. There was no statistically significant difference across gender (*Sig.* = 0.286) or total DAS score (*Sig.* = 0.816). The pro-conventional anesthesia students were significantly the most anxious while waiting for the drill to get ready (*Sig.* = 0.052). The pro-computer-controlled anesthesia students were the least anxious during periodontal instruments preparation (*Sig.* = 0.002) (Table 4).

**Table 4:** Students’ feedback of the anesthetic injections methods (*n* = 62)

Variable	Outcome	Computer-controlled (*n* = 26)	Conventional (*n* = 28)	Equal (*n* = 8)	*Sig.*
Gender	Female	20 (47.6%)	16 (38.1%)	6 (14.3%)	0.286
	Male	6 (30%)	12 (60%)	2 (10%)	
DAS	1st Domain	1.91 ± 0.74	1.79 ± 0.57	1.75 ± 0.46	0.755
Domain	2nd Domain	1.32 ± 0.47	1.07 ± 0.26	1.25 ± 0.46	0.085
	3rd Domain	1.46 ± 0.56	1.93 ± 0.81	1.50 ± 0.53	**0.052**
	4th Domain	1.00 ± 0	1.21 ± 0.42	1.50 ± 0.53	**0.002**
DAS total	(4–20)	5.77 ± 1.61	5.93 ± 1.41	5.75 ± 1.58	0.816

Bold values are significant ≤ 0.05

Chi-squared (*χ*^*2*^) test and Kruskal-Wallis (*H*) test used was a significance level (*Sig.*) ≤ 0.05

### Pain Perception on puncture (PPP)

In total, 45 (37.5%) of the administered injections were perceived with no pain on the puncture, 49 (40.8%) with slight pain, 23 (19.2%) with moderate pain, and 3 (2.5%) with severe pain. There were no statistically significant differences between females versus males (*Sig.* = 0.117), chronically-ill versus medically-free students (*Sig.* = 0.426), medications recipients versus non-recipients (*Sig.* = 0.314) or smoking versus non-smoking students (*Sig.* = 0.339). In contrast, the difference between the injections administered by the students and experienced dentist was statistically significant (*Sig.* = 0.016), with less pain experienced when administered by the dentist. Similarly, the infiltration technique was significantly associated (*Sig.* = 0.014) with less pain than the nerve block technique (Table 5).

**Table 5:** Levels of pain perception on puncture of anesthetic injections (*n* = 120)

Variable	Outcome	No Pain	Slight Pain	Moderate Pain	Severe Pain	*Sig.*
Gender	Female	34 (41.5%)	35 (42.7%)	11 (13.4%)	2 (2.4%)	0.117
	Male	11 (28.9%)	14 (36.8%)	12 (31.6%)	1 (2.6%)	
Chronic	Yes	4 (33.3%)	4 (33.3%)	3 (25%)	1 (8.3%)	0.426
Illness	No	41 (38%)	45 (41.7%)	20 (18.5%)	2 (1.9%)	
Medical	Yes	5 (41.7%)	3 (25%)	3 (25%)	1 (8.3%)	0.314
Treatment	No	40 (37%)	46 (42.6%)	20 (18.5%)	2 (1.9%)	
Smoking	Yes	1 (12.5%)	4 (50%)	3 (37.5%)	0 (0%)	0.339
Frequency	No	44 (39.3%)	45 (40.2%)	20 (17.9%)	3 (2.7%)	
Operator	Student	18 (29%)	24 (38.7%)	17 (27.4%)	3 (4.8%)	0.016*
	Dentist	27 (46.6%)	25 (43.1%)	6 (10.3%)	0 (0%)	
Injection	Infiltration	39 (43.8%)	36 (40.4%)	13 (14.6%)	1 (1.1%)	0.014*
Technique	Nerve Block	6 (19.4%)	13 (41.9%)	10 (32.3%)	2 (6.5%)	
Injection	Computer-controlled	22 (36.7%)	24 (40%)	14 (23.3%)	0 (0%)	0.285
Method	Conventional	23 (38.3%)	25 (41.7%)	9 (15%)	3 (5%)	

Bold values are significant ≤ 0.05

Fisher’s Exact Test was used as a significance level (*Sig.*) ≤ 0.05

**Fig. 3:**
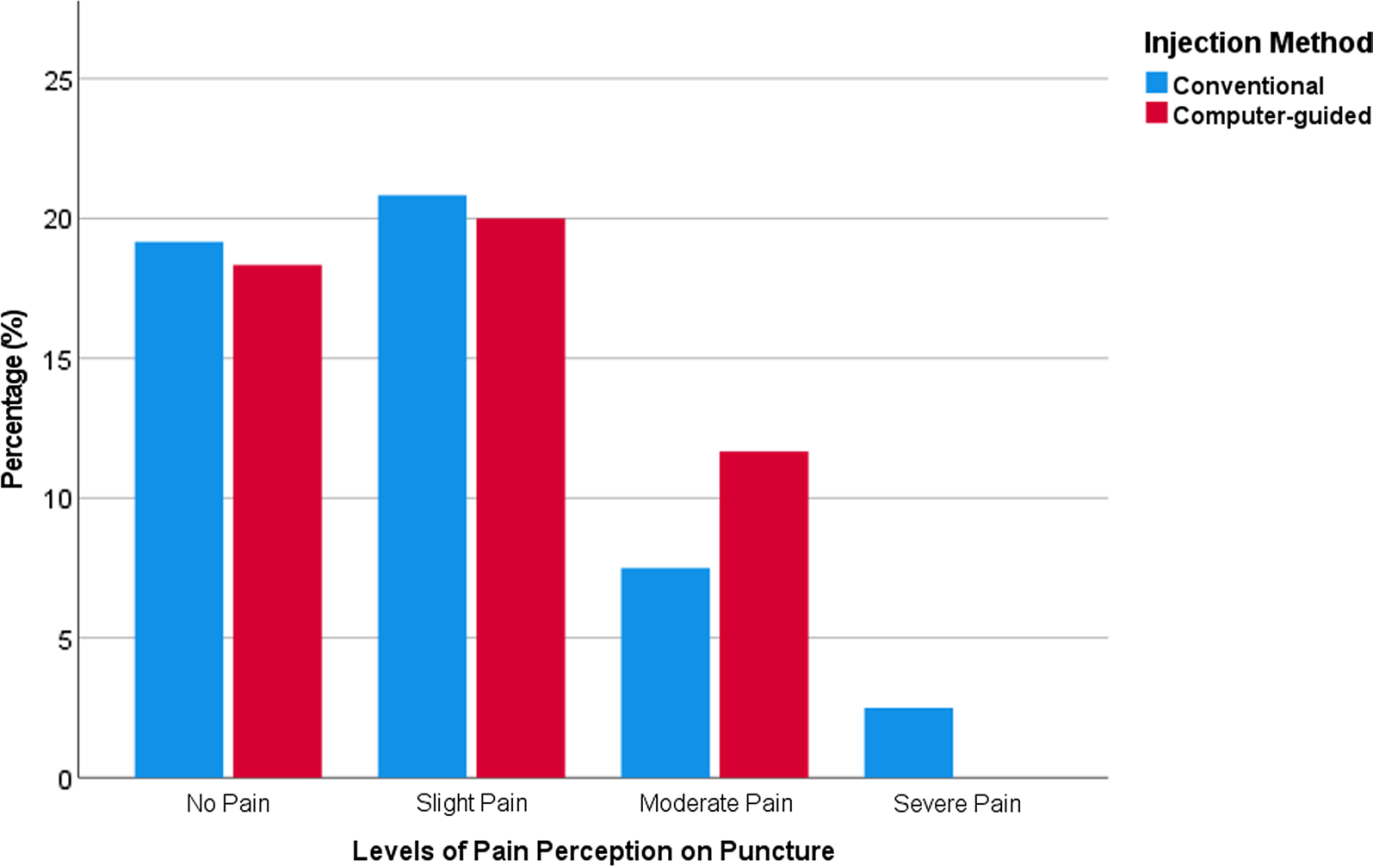
Levels of perceived pain on puncture stratified by injection method (n = 120)

The difference between computer-controlled and conventional injections was not statistically significant (*Sig.* = 0.285). In the computer-controlled group, 22 (36.7%) were without pain, 24 (40%) with slight pain, 14 (23.3%) with moderate pain, and 0 (0%) with severe pain. In the conventional group, 23 (38.3%) were without pain, 25 (41.7%) with slight pain, 9 (15%) with moderate pain, and 3 (5%) with severe pain (Fig. 3)

On evaluating the perceived pain on puncture using VAS, there were no statistically significant differences across gender (*Sig.* = 0.133), chronic illnesses (*Sig.* = 0.706), medical treatments (*Sig.* = 0.352), and smoking (*Sig.* = 0.256). The difference between computer-controlled (1.33 ± 1.59) and conventional injections (1.81 ± 2.03) was not statistically significant (*Sig.* = 0.213) (Table 6).

**Table 6:** VAS of pain perception on puncture (*n* = 120)

Variable	Outcome	VAS Score	*Sig.*
Gender	Female	1.46 ± 1.81	0.133
	Male	1.81 ± 1.88	
Chronic illnesses	Yes	1.55 ± 1.80	0.706
	No	1.75 ± 2.22	
Medical treatments	Yes	2.18 ± 2.31	0.352
	No	1.50 ± 1.77	
Smoking	Yes	2.40 ± 2.31	0.256
	No	1.51 ± 1.79	
Operator	Student	2.12 ± 2.03	**< 0.001**
	Dentist	0.98 ± 1.39	
Injection technique	Infiltration	1.21 ± 1.60	**< 0.001**
	Nerve Block	2.58 ± 2.09	
Injection method	Computer-controlled	1.33 ± 1.59	0.213
	Conventional	1.81 ± 2.03	

Bold values are significant ≤ 0.05

Mann-Whitney (*U*) test was used was a significance level (*Sig.*) ≤ 0.05

**Fig. 4:**
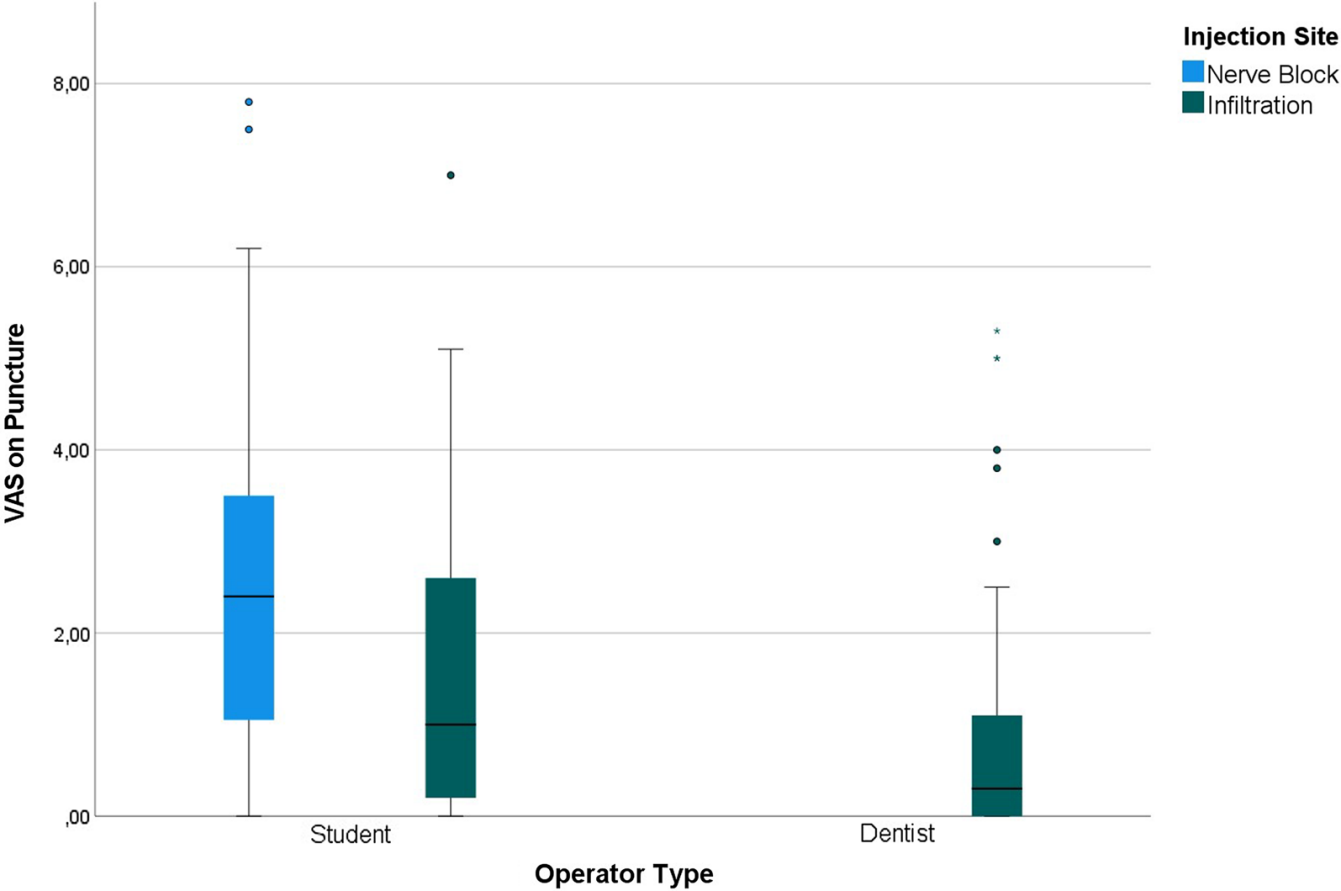
VAS on puncture stratified by operator and injection technique (n = 120)

The student-administered injections (2.12 ± 2.03) and nerve block injections (2.58 ± 2.09) had significantly higher VAS scores than the dentist-administered injections (0.98 ± 1.39) and infiltration injections (1.21 ± 1.60), respectively (Fig. 4).

### Pain perception during injection (PPI)

Evaluation of perceived pain during injection using VAS revealed that there were no statistically significant differences across gender (*Sig.* = 0.509), chronic illnesses (*Sig.* = 0.476), medical treatments (*Sig.* = 0.613), and smoking (*Sig.* = 0.230) (Table 7).

**Table 7:** VAS of pain perception during injection (*n* = 120)

Variable	Outcome	VAS Score	*Sig.*
Gender	Female	2.19 ± 2.25	0.509
	Male	1.82 ± 1.96	
Chronic illness	Yes	2.13 ± 2.83	0.476
	No	2.07 ± 2.09	
Medical treatment	Yes	2.18 ± 2.02	0.613
	No	2.06 ± 2.18	
Smoking	Yes	3.44 ± 3.01	0.230
	No	1.97 ± 2.07	
Operator	Student	2.49 ± 2.26	**0.009**
	Dentist	1.63 ± 1.97	
Injection technique	Infiltration	1.78 ± 1.89	**0.026**
	Nerve Block	2.92 ± 2.65	
Injection method	Computer-controlled	1.65 ± 1.93	**0.029**
	Conventional	2.49 ± 2.31	

Bold values are significant ≤ 0.05

Mann-Whitney (*U*) test was used was a significance level (*Sig.*) ≤ 0.05

**Fig. 5:**
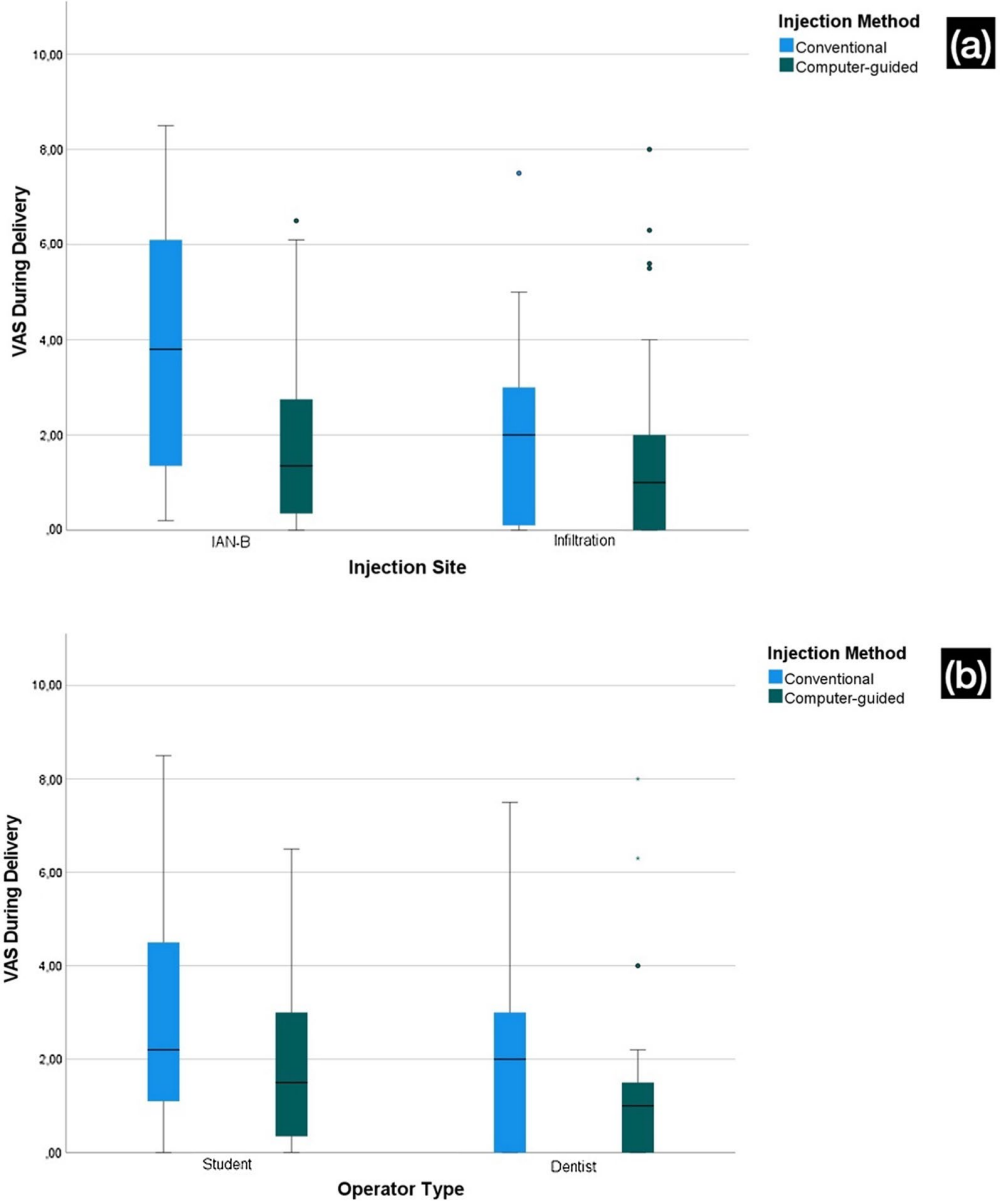
VAS during Injection Stratified by Operator, Injection Technique and Injection Method (n = 120). **a** VAS during Injection Stratified by Injection Method and Injection Technique. **b** VAS during Injection Stratified by Injection Method and Operator

The student-administered injections (2.49 ± 2.26) and nerve block injections (2.92 ± 2.65) had significantly higher VAS scores than the dentist-administered injections (1.63 ± 1.97) and infiltration injections (1.78 ± 1.89), respectively. The difference between computer-controlled (1.65 ± 1.93) and conventional injections (2.49 ± 2.31) was statistically significant (*Sig.* = 0.029) (Fig. 5).

### Dental anxiety scale (DAS)

Female students (6.17 ± 1.57) had a significantly (*Sig.* = 0.030) higher DAS score compared to their male counterparts (5.53 ± 1.56). Total DAS score was not significantly different between chronically-ill versus medically-free students (*Sig.* = 0.510), medications recipients versus non-recipients (*Sig.* = 0.336) or smoking versus non-smoking students (*Sig.* = 0.534).

Similarly, total DAS was not significantly different between student-administered versus dentist-administered injections (*Sig.* = 0.454), infiltration versus nerve block injections (*Sig.* = 0.669), or computer-controlled versus conventional injections (*Sig.* = 1.000) (Table 8).

**Table 8:** Dental anxiety scale (DAS) score of the participating students (n = 120)

Variable	Outcome	DAS Score	*Sig.*
Gender	Female	6.17 ± 1.57	**0.030**
	Male	5.53 ± 1.56	
Chronic illness	Yes	6.17 ± 1.40	0.510
	No	5.94 ± 1.61	
Medical treatment	Yes	6.33 ± 1.56	0.336
	No	5.93 ± 1.59	
Smoking	Yes	5.50 ± 0.93	0.534
	No	6.00 ± 1.62	
Operator	Student	5.84 ± 1.50	0.454
	Dentist	6.10 ± 1.68	
Injection technique	Infiltration	6.01 ± 1.51	0.669
	Nerve Block	5.84 ± 1.62	
Injection method	Computer-controlled	5.97 ± 1.59	1.000
	Conventional	5.97 ± 1.59	

Bold value is significant ≤ 0.05

Mann-Whitney (*U*) test was used was a significance level (*Sig.*) ≤ 0.05

Using Spearman’s rank coefficient, the bivariate correlation revealed that VAS on puncture and VAS during injection had a moderate correlation (*ρ* = 0.449; *Sig.* < 0.001). On the other hand, the total DAS score was not correlated with either VAS on puncture or VAS during the injection (Table 9)

**Table 9:** Correlation of dental anxiety scale (DAS) and the VAS of pain perception (*n* = 120)

		VAS on Puncture	VAS during Injection	Total DAS Score
VAS on puncture	*ρ*	1.000	0.449	0.078
	Sig.		**< 0.001**	0.398
VAS during injection	*ρ*	0.449	1.000	0.040
	Sig.	**< 0.001**		0.666
Total DAS score	*ρ*	0.078	0.040	1.000
	Sig.	0.398	0.666	

Bold values are significant ≤ 0.05

Spearman’s rank correlation (*ρ*) was used was a significance level (*Sig.*) < 0.05

## Discussion

Dental phobia is one of the main barriers which make patients avoid routine check-ups and early treatments [39]. Indeed, patients seek to avoid pain caused by dental procedures [40] For pain prevention or reduction, profound dental anesthesia is required. Nevertheless, LA is also related to pain sensation due to the injection and application, which makes this problem unsolvable [41]. Consequently, many new devices and techniques were developed to minimise pain during the application of local dental anesthesia [42]. CCLA is one of these attempts and many commercially available instruments [43, 44]. This study aimed to evaluate and compare the pain sensation resulting from using conventional and CCLA.

The study sample included a majority of females (68.3%), medically-free (90%) and non-smokers. These results are associated with the study subject selection as they are all dental students. Dental students as a study subject to test two different LA methods offers several advantages. Participants’ homogeneity, better evaluation of the pain sensation and giving the future dentists the opportunity to have their own experience with the two LA methods are among other the most essential reasons for choosing the study group. The investigation in the study included regarding the operator two groups, the “students” group in which dental students performed all anesthesia to dental students as a part of the local anesthesia course [45] and the “dentist” where an experienced oral surgeon was the operator.

On evaluating the ease of handling, 54% of the participating students rated the conventional syringe as easier, while 41.9% preferred the computer-controlled and 12.9% rated both equally. It is also worth mentioning that each subject found the signal tone of the computer-assisted Calaject^®^ system disturbing during treatment. This disturbance caused a further increase in the stress level of some subjects and thus presumably a subjective increase in the perception of pain. Therefore, it is advised to reduce the signal tone on the volume button to a minimum.

Pain perception on puncture was assessed in both groups. We used in all interventions the same needle (Transcodent^®^ painless needle) which was proved to produce less pain than the regular needle tips [46]. The difference between the injections administered by the students and the experienced dentist was statistically significant (*Sig*. = 0.016) with a trend towards lesser pain experienced after the dentist-administered injections. The student group performed the LA technique for the first time on their peers [47] whereas the dentist had performed numerous LA shots throughout his 15 years of practice. This may clarify the differences between the two groups.

Similarly, the infiltration technique was significantly associated (*Sig*. = 0.014) with less pain than the nerve block technique. This result is in accordance with findings reported that infiltrations were less painful than inferiors alveolar nerve block technique [48, 49]. Additionally, pain perception on puncture between computer-controlled and conventional injections was not statistically significant (Sig. = 0.285). This seems to be a logical result as the same needle was used in both techniques. A similar result was reported by Flisfisch et al. by testing computer assistant LA versus conventional one; there was no difference in the mean sensation of mucosal puncture [50].

Pain perception during the injection was evaluated in both groups and techniques. The student-administered injections (2.49 ± 2.26) and nerve block injections (2.92 ± 2.65) had significantly higher VAS scores than the dentist-administered injections (1.63 ± 1.97) and infiltration injections (1.78 ± 1.89), respectively. Also, here the operator’s experience seems to play an essential role in the pain reduction during the injection [19].

The difference in pain sensation during the injection between the computer-controlled (1.65 ± 1.93) and conventional injections (2.49 ± 2.31) was less painful for the computer-controlled method and statistically significant (*Sig*. = 0.029). Computer-controlled devices, which control the administered anesthetic rate to the soft tissues are used to lessen discomfort during local anesthesia for dental procedures [51]. Several studies with similar study designs conclude pain reduction with using a computer-assisted device [50, 52, 53]. Although, both operators of the study (dentist and students) were trained to administrate the LA very slowly, the computer-controlled devices still applied slower and less painful injections [43]. This notion underscores the importance of keeping a consistently slow administration the LA to reduce the pain perception [42, 54, 55].

On the other hand, dental practice requires both clinically-effective and time-efficient treatments; therefore, injections with higher speed are more likely to be used [56]. In this case it is recommended to use computer-controlled devices [57]. One advantage of using the CCLA system is the self-aspiration function. The device performs this automatically before the start of the injection. This significantly reduces the risk of accidental intravascular injection. Intra-arterial injection of the LA includes vasoconstrictor, which can lead to severe complications such as permanent blindness [58], cardiovascular and central nervous system toxicity [59, 60]. In the hectic of the everyday professional life a forgotten aspiration can cause a severe complication which is unlikely to occur if a computer-controlled LA device is used.

In the study sample, female students had a significantly higher DAS score compared to their male counterparts. Several studies investigating dental anxiety levels across the gender groups came to the same conclusion [61–63]; however, other studies reported no significant differences between the gender [64, 65]. The high anxiety level of females might be due to the gender-specific socialization patterns related to pain beliefs, expectations, and subsequent behaviors [66, 67]. Unlike previous studies, DAS was not significantly different between student-administered versus dentist-administered injections (*Sig.* = 0.454), infiltration versus nerve bloc injections (*Sig.* = 0.669), or computer-controlled versus conventional injections (*Sig.* = 1.000). These findings should be interpreted with caution, as DAS may not have been an appropriate tool for this type of clinical trials [68].

### Strengths

This study compares the effectiveness of the CCLA devices in pain reduction in clinical trial in which the study subjects have an adequate homogeneity as they were all dental students and at the same time also the operators. This was possible as the study conducted as a part of the dental local anesthesia course for the sixth semester dental students. In addition, the two LA methods were used by an experienced dentist in addition to the dental students to examine in a split-mouth design whether the dentist’s experience influenced pain reduction when using LA.

### Limitations

The first limitation of this study is the small sample size, which can be justified by the fact that this size was calculated based on the assumption of 5% margin error; therefore, future studies should consider lower margin errors when comparing computer-controlled versus conventional LA. The second limitation is the controlled environment where this study was conducted, which may reduce the generalizability of the findings. Objective feedback from operators with an appropriately detailed questionnaire should be used for future studies. The Periodontal status of the study subjects was not assessed. In further studies should, the periodontal status in relation to the pain response should be investigated [69, 70].

## Conclusion

CCLA is less painful when applying the injection than the conventional syringe, even though the two methods’ pain perception on puncture was not significantly different. Sex, chronic illnesses, medications, and smoking were not predictors of pain perception. Clinical experience seems to help in reduction of pain perception. Therefore, the dentist-administered injections were less painful compared to the student-administered injections.

## Data Availability

The datasets used and/or analysed during the current study are available from the corresponding author on reasonable request. For privacy reasons, however, individual data allowing for the identification of participants cannot be made available.

## Abbreviations

LA: Local anesthesia
CCLA: Computer-controlled local anesthesia
RCT: Randomised controlled trial
CONSORT: Consolidated Standards of Reporting Trials
VAS: Visual analog scale
DAS: Dental Anxiety Scale
SPSS: Statistical Package for the Social Sciences
*µ*: Mean
*SD*: Standard deviation
*CI*: Confidence level
*Sig.*: Significance level
